# Comparative Classification Performance of Anthropometric, Body Composition, and Cardiometabolic Indices for Identifying ALAD-Defined Metabolic Syndrome in Panamanian Adults

**DOI:** 10.3390/medsci14040500

**Published:** 2026-08-20

**Authors:** Griselda Arteaga, Xenia Hernandez Adames, Ivonne Torres-Atencio, Ana Espinosa De Ycaza, Maria Fabiana Piran Arce, Ana Tejada Espinosa, Orlando Serrano Garrido

**Affiliations:** 1 Department of Human Microbiology, Faculty of Medicine, University of Panama, Panama City 0801, Panama; griselda.arteaga@up.ac.pa; 2 Faculty of Medicine, Department of Biochemistry and Nutrition, Universidad de Panamá, Panama City 0801, Panama; xeniaupma@gmail.com (X.H.A.); ana.tejadae@up.ac.pa (A.T.E.); 3 Faculty of Medicine, Department of Pharmacology, Universidad de Panamá, Panama City 0801, Panama; ivonne.torres@up.ac.pa; 4 Sistema Nacional de Investigación (SNI), SENACYT, Edificio 205, Ciudad del Saber, Clayton, Panama City 0801, Panama; 5 Faculty of Medicine, Department of Medicine, Universidad de Panamá, Panama City 0801, Panama; anaelena.espinosa@up.ac.pa; 6 Faculty of Health Sciences, Universidad Nacional de San Luis, San Luis 5700, Argentina; mpiran@unsl.edu.ar

**Keywords:** cardiometabolic index, lipid accumulation product, atherogenic index of plasma, waist-to-height ratio, homeostasis model assessment of insulin resistance

## Abstract

Background: Composite anthropometric and cardiometabolic indices have emerged as practical tools for identifying metabolic syndrome (MetS), but their comparative classification performance against ALAD-defined MetS has not been comprehensively evaluated in Panamanian adults. This study compared the classification performance of selected anthropometric, body composition, and cardiometabolic indices for identifying metabolic syndrome defined according to the Latin American Diabetes Association (Asociación Latinoamericana de Diabetes [ALAD]) criteria in non-diabetic Panamanian adults. Methods: This cross-sectional study included 262 adults without diabetes (110 men and 152 women). Clinical and laboratory measurements were used to calculate the body mass index (BMI), waist-to-height ratio (WHtR), visceral adiposity index (VAI), lipid accumulation product (LAP), triglyceride-to-high-density lipoprotein cholesterol ratio (TG/HDL-C), atherogenic index of plasma (AIP), cardiometabolic index (CMI), and Homeostasis Model Assessment of Insulin Resistance (HOMA-IR). Classification performance was evaluated using receiver operating characteristic (ROC) curve analysis, optimal cut-off values, and DeLong’s test. Age-adjusted logistic regression assessed associations between each index and ALAD-defined MetS. Results: Composite indices integrating anthropometric and metabolic parameters showed high classification performance. CMI had the highest observed AUC in men (AUC = 0.933; 95% CI: 0.887–0.979), whereas LAP had the highest observed AUC in women (AUC = 0.854; 95% CI: 0.778–0.931). Conclusions: CMI and LAP showed high classification performance for identifying ALAD-defined MetS in Panamanian adults without diabetes, with the highest observed AUCs in men and women, respectively. These simple, inexpensive indices may have potential utility as screening tools for identifying ALAD-defined MetS in primary healthcare settings. The proposed cut-off values are exploratory and require external validation in independent Panamanian and Latin American populations before clinical implementation.

## 1. Introduction

Metabolic syndrome (MetS) is a cluster of cardiometabolic risk factors characterized by central obesity, atherogenic dyslipidemia, hypertension, hyperglycemia, and a proinflammatory and prothrombotic state, all of which substantially increase the risk of cardiovascular disease, type 2 diabetes mellitus (T2DM), chronic kidney disease, and premature mortality [1]. MetS has become a major public health challenge, with an estimated prevalence of approximately 40% in Latin America, influenced by demographic factors such as age, sex, and urbanization [2,3]. In Panama, data from the National Health Survey (ENSPA 2019) indicate that more than 67% of the adult population has overweight or obesity, underscoring the growing burden of cardiometabolic disease in the country [4].

In Panama, cardiovascular disease remains the leading cause of death, accounting for more than 20% of all annual deaths according to the National Institute of Statistics and Census [5]. Diabetes is the third leading cause of morbidity and mortality, with a national prevalence of 14.4% [4], while chronic kidney disease affects more than 10% of the population and is largely driven by hypertension and diabetes mellitus [6]. Recent evidence from Panama highlights the growing cardiometabolic burden in populations at increased risk. A recent study among adults living with HIV reported a high prevalence of metabolic syndrome and its cardiometabolic components, emphasizing the need for earlier screening and improved cardiometabolic risk assessment strategies in the country [7]. Early identification and appropriate management of MetS are therefore essential, emphasizing the need for simple, accurate, and cost-effective screening tools that can facilitate timely cardiometabolic risk assessment, particularly in populations at increased cardiometabolic risk [8].

In this context, there is a clear need for accessible, reproducible, and cost-effective tools for the early identification of individuals at increased cardiometabolic risk. Several composite cardiometabolic indices integrating anthropometric and biochemical measurements have been proposed as practical alternatives for cardiometabolic risk assessment. By combining complementary information on body fat distribution and lipid metabolism, these indices may better capture the complex pathophysiological mechanisms underlying metabolic syndrome than isolated anthropometric or biochemical markers [9]. Among the most extensively studied composite indices are the Cardiometabolic Index (CMI), Lipid Accumulation Product (LAP), Visceral Adiposity Index (VAI), triglyceride-to-high-density lipoprotein cholesterol ratio (TG/HDL-C), and Atherogenic Index of Plasma (AIP), which have been investigated in relation to metabolic syndrome and other cardiometabolic conditions across diverse populations [9,10,11,12,13,14]. Because these indices rely on routinely available clinical and laboratory parameters, they may represent practical tools for cardiometabolic screening in primary healthcare settings. Although the Homeostasis Model Assessment of Insulin Resistance (HOMA-IR) remains a widely used surrogate marker of insulin resistance, its routine clinical application is limited because fasting insulin measurements are relatively costly and are not routinely available in many primary healthcare settings [14].

Among the available composite cardiometabolic indices, the CMI and LAP have shown promising performance for identifying metabolic syndrome in different populations. Comparative studies have reported favorable discriminatory capacity for these indices relative to traditional anthropometric indicators, including sex-specific analyses [15,16]. Other indices, including the Visceral Adiposity Index (VAI), triglyceride-to-high-density lipoprotein cholesterol ratio (TG/HDL-C), and Atherogenic Index of Plasma (AIP), have also been investigated in relation to metabolic syndrome and other cardiometabolic conditions, with performance varying according to population characteristics and the criteria applied [9,12,13]. Collectively, these findings support the evaluation of cardiometabolic indices within specific populations before considering their broader application in screening or clinical settings.

Despite the growing body of evidence supporting the use of composite cardiometabolic indices for metabolic syndrome screening, their comparative classification performance against defined metabolic syndrome criteria has not been comprehensively evaluated in the Panamanian adult population. Because the accuracy of these indices may be influenced by ethnic composition, body fat distribution, lifestyle, and the diagnostic criteria used to define metabolic syndrome, findings from other populations may not be directly generalizable to the Panamanian population [9,12,15]. Therefore, evaluation in the local population is essential to identify the most appropriate indices and derive exploratory sex-specific cut-off values for subsequent external validation.

## 2. Materials and Methods

### 2.1. Study Design and Participants

**Figure 1:**
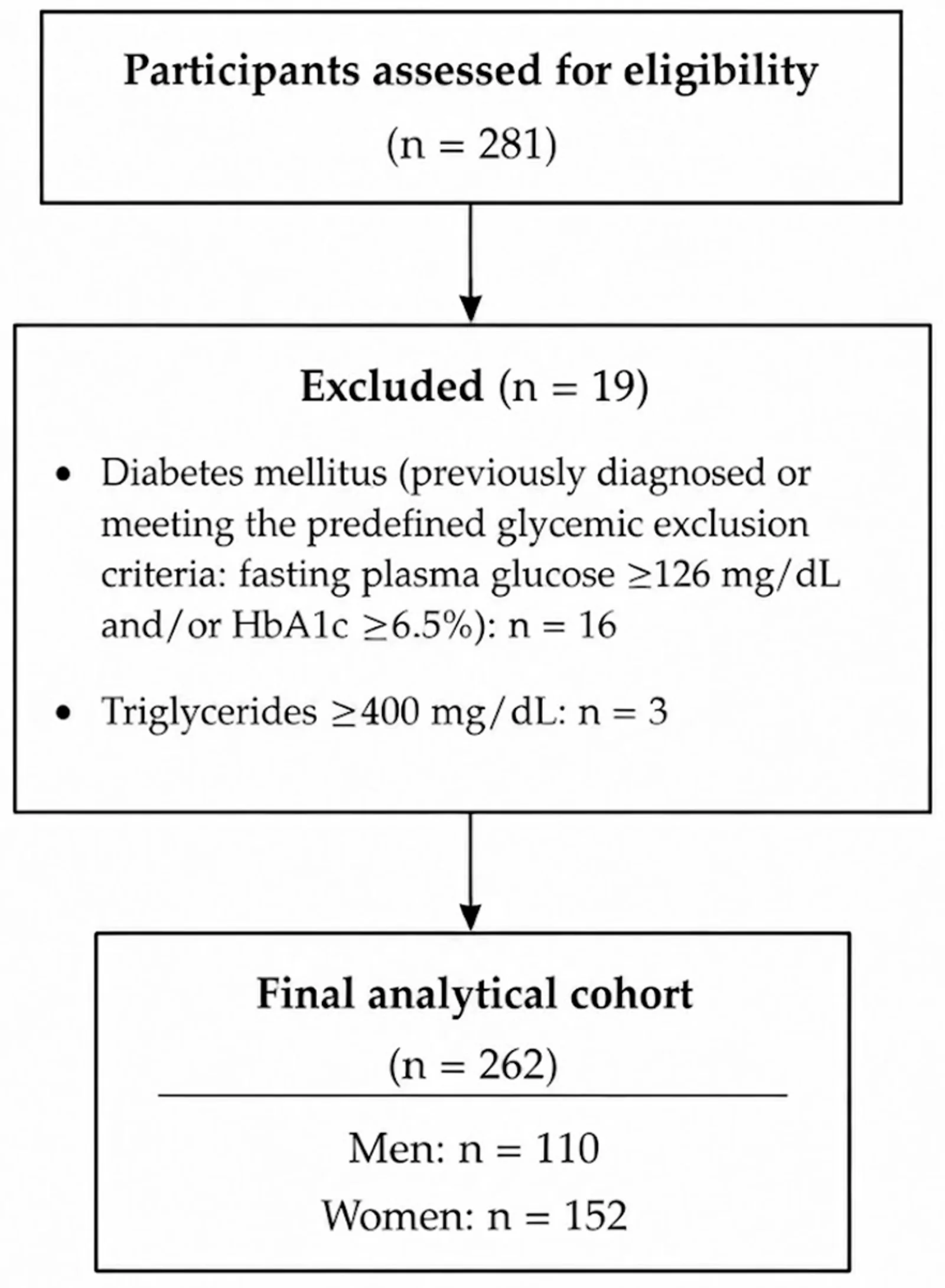
Participant flow and derivation of the final analytical cohort.

This cross-sectional study was conducted among adults residing in urban areas of the provinces of Panama and Panama Oeste between October 2022 and November 2023. A public invitation was disseminated through social media to recruit men and women aged 18–60 years, who were evaluated after providing written informed consent at the Immunology Laboratory and the Department of Biochemistry and Nutrition of the University of Panama. Eligible participants underwent clinical, anthropometric, body composition, and biochemical assessments following an 8–12 h fasting period. Exclusion criteria included age <18 or >60 years; previously diagnosed type 2 diabetes mellitus, regardless of glycemic control, or newly identified diabetes defined by fasting plasma glucose ≥ 126 mg/dL and/or HbA1c ≥ 6.5% [17]; triglyceride concentrations ≥400 mg/dL; pregnancy or lactation; use of systemic corticosteroids or other medications known to substantially affect lipid metabolism; and incomplete records. A total of 281 participants were assessed for eligibility. After applying the predefined eligibility criteria, 19 participants were excluded: 16 because of previously diagnosed type 2 diabetes mellitus or fulfillment of the predefined glycemic exclusion criteria (fasting plasma glucose ≥126 mg/dL and/or HbA1c ≥ 6.5%), and three because of triglyceride concentrations ≥400 mg/dL. The final analytical cohort therefore comprised 262 participants (110 men and 152 women), as summarized in Figure 1. Statistical analyses were performed using complete-case data for each statistical procedure; therefore, the number of participants included varied slightly according to the availability of the variables required for each analysis. Participants were classified as having or not having metabolic syndrome according to the criteria of the Latin American Diabetes Association (Asociación Latinoamericana de Diabetes [ALAD]) [18]. The components and thresholds used for this classification are described in Section 2.4.

### 2.2. Clinical Questionnaire and Anthropometric Measurements

Each participant completed a standardized medical questionnaire that collected information on previous diagnoses of diabetes and hypertension, current medication use, physical activity, and peri- and menopausal status in women. Blood pressure was measured in both arms using an automated brachial sphygmomanometer after participants had remained seated for a resting period [19]. Anthropometric measurements included body weight, height, waist circumference, hip circumference, and neck circumference [20,21]. Body composition was assessed by bioelectrical impedance analysis using a MC-580 body composition analyzer (Tanita Corporation, Tokyo, Japan). Physical activity was classified into three categories (low, moderate, and high) using the classification included in the study questionnaire, which was adapted from the categories proposed in the International Physical Activity Questionnaire (IPAQ) [22]. For the analyses evaluating the association between physical activity and metabolic syndrome, the moderate- and high-activity categories were combined as sufficient physical activity, whereas the low-activity category was considered insufficient physical activity.

### 2.3. Laboratory Measurements

Venous blood samples were collected after an 8–12 h fasting period into serum and EDTA tubes for biochemical analyses. Fasting plasma glucose, total cholesterol, triglycerides, and high-density lipoprotein cholesterol (HDL-C) were measured using enzymatic colorimetric methods on a Chemray 240 automated biochemical analyzer (Rayto Life and Analytical Sciences Co., Ltd., Shenzhen, China) Low-density lipoprotein cholesterol (LDL-C) and very-low-density lipoprotein cholesterol (VLDL-C) were calculated. Fasting insulin concentrations were determined by chemiluminescence using the Flash 1200 chemiluminescence immunoassay analyzer (Shenzhen YHLO Biotech Co., Ltd., Shenzhen, China), and glycated hemoglobin (HbA1c) was measured by high-performance liquid chromatography (HPLC) using the H8 hemoglobin analyzer (Lifotronic Technology Co., Ltd., Shenzhen, China)**.** All biochemical measurements were performed following the manufacturers’ recommendations using the corresponding commercial reagents, calibrators, and internal quality controls.

### 2.4. Definition of Metabolic Syndrome

Participants were classified as having or not having metabolic syndrome according to the criteria of the Latin American Diabetes Association (Asociación Latinoamericana de Diabetes [ALAD]) [18]. For the present study, metabolic syndrome was defined as the presence of central obesity together with at least two additional metabolic abnormalities. The ALAD components and thresholds applied for metabolic syndrome classification are presented in Table 1. Antihypertensive treatment was considered sufficient to classify the blood-pressure component as positive, regardless of the blood pressure measured at the study visit. Current use of lipid-lowering medication was considered when defining the lipid component of MetS; however, the specific indication for lipid-lowering therapy (e.g., treatment specifically prescribed for hypertriglyceridemia) was not available and therefore was not used as an additional criterion. Although medication information was collected, review of the analytical dataset identified no participant with documented pharmacological treatment specifically prescribed for hypertriglyceridemia. Therefore, lipid-lowering medication was not used as an additional criterion for classification of the triglyceride component.

### 2.5. Cardiometabolic Indices

The evaluated anthropometric, body composition, and cardiometabolic indices included the Homeostasis Model Assessment of Insulin Resistance (HOMA-IR), triglyceride-to-high-density lipoprotein cholesterol ratio (TG/HDL-C), atherogenic index of plasma (AIP), waist-to-height ratio (WHtR), cardiometabolic index (CMI), lipid accumulation product (LAP), visceral adiposity index (VAI), body mass index (BMI), and the body composition marker visceral fat rating (VFR). The formulas and original references used to calculate each index are presented in Table 2. For indices requiring lipid concentrations expressed in mmol/L, triglyceride and HDL-C values were converted from mg/dL to mmol/L before calculation according to the units specified in the original published formulas. The AIP was calculated as log10(TG/HDL-C) using triglyceride and HDL-C concentrations expressed in mmol/L. This index should not be confused with the traditional atherogenic index (AI; total cholesterol/HDL-C), which was not evaluated in the present study. Because no validated HOMA-IR threshold is currently available for Panamanian adults, the sex-specific HOMA-IR cut-off values derived in this study should be considered exploratory.

Because several evaluated indices incorporate anthropometric and biochemical variables that are also components of the ALAD definition of metabolic syndrome, the ROC analyses were interpreted as measures of classification performance against ALAD-defined MetS rather than as evidence of independent diagnostic ability. This potential mathematical overlap was considered when interpreting the magnitude of the observed AUC values. All cut-off values derived in the present study should be considered exploratory because they were derived and evaluated within the same study population and require validation in independent populations.

### 2.6. Statistical Analysis

The distribution of continuous variables was assessed using the Shapiro–Wilk test. Continuous variables are presented as medians and interquartile ranges (IQRs) and were compared between participants with and without metabolic syndrome using the Mann–Whitney U test. Categorical variables are presented as frequencies and percentages and were compared using the chi-square test or Fisher’s exact test, as appropriate.

The classification performance of each anthropometric, body composition, and cardiometabolic index for identifying ALAD-defined metabolic syndrome was evaluated using receiver operating characteristic (ROC) curve analysis. The area under the ROC curve (AUC) with 95% confidence intervals (95% CIs) was calculated, and the optimal cut-off value for each index was determined by maximizing the Youden index. Sensitivity, specificity, positive predictive value (PPV), and negative predictive value (NPV) were subsequently calculated using the selected cut-off values. Differences between correlated ROC curves were assessed using the DeLong test. Pairwise DeLong comparisons were not adjusted for multiple testing; therefore, the resulting *p* values were considered exploratory and interpreted accordingly.

Internal validation of the ROC-derived cut-off values was performed using 2000 stratified bootstrap resamples separately within each sex and stratified by ALAD-defined metabolic syndrome status, thereby preserving the number of participants with and without metabolic syndrome in each resample. For each index, resampling was restricted to participants with available data for that index. In each bootstrap sample, the optimal cut-off was re-estimated using the Youden index. Percentile-based 95% confidence intervals were obtained from the distribution of bootstrap estimates to assess the stability and uncertainty of the derived cut-off values. Sensitivity and specificity were internally evaluated using out-of-bag observations not selected in each bootstrap resample. All internally derived cut-off values were considered exploratory and require external validation before clinical implementation. Detailed bootstrap validation results are presented in Supplementary Table S1.

Binary logistic regression analyses were performed to evaluate the association between each anthropometric, body composition, and cardiometabolic index and ALAD-defined metabolic syndrome. Because several indices share common anthropometric and biochemical components, each index was evaluated in a separate regression model rather than being entered simultaneously into a single model. Models were adjusted for age and fitted for the overall study population and separately for men and women. Odds ratios (ORs) with 95% confidence intervals (95% CIs) were estimated. Variables expressed on markedly different numerical scales (e.g., AIP and WHtR) were rescaled before analysis to facilitate interpretation of the estimated ORs.

As a complementary exploratory analysis, separate multivariable logistic regression models were fitted for each index in the overall sample, additionally adjusting for age, sex, and physical activity. These covariates were selected a priori based on their potential association with metabolic syndrome and availability in the study population. Complete-case analysis was used for each model. Results are presented in Supplementary Table S2.

### 2.7. Ethical Considerations

The study was conducted in accordance with the principles of the Declaration of Helsinki and was approved by the Bioethics Committee of the University of Panama (CBUP) under approval codes CBUP/588/2021 (15 December 2021) and CBUP/101/2022 (1 April 2022). Written informed consent was obtained from all participants prior to enrollment.

## 3. Results

A total of 262 participants (152 women and 110 men) were included in the final analytical cohort after applying the predefined eligibility criteria. The prevalence of metabolic syndrome was 15.8% (24/152) among women and 24.5% (27/110) among men. Clinical, biochemical, anthropometric, and cardiometabolic characteristics according to metabolic syndrome status are presented in Table 3. Differences between participants with and without metabolic syndrome were observed across several clinical, biochemical, anthropometric, and cardiometabolic variables, with statistical significance varying according to sex and the variable evaluated.

The classification performance of the evaluated cardiometabolic and anthropometric indices against ALAD-defined metabolic syndrome (MetS) is presented in Table 4. Receiver operating characteristic (ROC) analyses were performed separately for men and women. The area under the ROC curve (AUC), optimal cut-off values, sensitivity, specificity, positive predictive value, and negative predictive value for each index are presented in Table 4.

Internal bootstrap validation showed variability in the precision of the sex-specific cut-off estimates, with wider confidence intervals observed for several indices. Out-of-bag estimates of sensitivity and specificity likewise showed variable precision across indices. Detailed bootstrap-derived cut-off values and performance estimates are presented in Supplementary Table S1.

Pairwise comparisons of correlated ROC curves using the DeLong test are presented in Table 5. In men, statistically significant differences were observed between CMI and TG/HDL-C ratio (*p* = 0.032), CMI and AIP (*p* = 0.032), and CMI and BMI (*p* = 0.023). In women, statistically significant differences were observed between LAP and VAI (*p* = 0.010), LAP and TG/HDL-C ratio (*p* = 0.027), and LAP and AIP (*p* = 0.027). No statistically significant differences were identified for the remaining pairwise comparisons, indicating that numerical differences in AUC should not be interpreted as statistically demonstrated superiority.

Age-adjusted binary logistic regression analyses were performed to evaluate the association between each index and ALAD-defined metabolic syndrome (Table 6). All evaluated anthropometric, body composition, and cardiometabolic indices were significantly associated with ALAD-defined metabolic syndrome in the overall sample and in sex-stratified analyses (all *p* < 0.05). The corresponding odds ratios (ORs), 95% confidence intervals (95% CIs), and *p* values are presented in Table 6.

In complementary exploratory models additionally adjusted for sex and physical activity, all evaluated indices remained significantly associated with ALAD-defined MetS, with effect estimates similar to those observed in the age-adjusted models (Supplementary Table S2).

The association between physical activity and ALAD-defined metabolic syndrome is presented in Table 7. Participants reporting sufficient physical activity had lower estimated odds of ALAD-defined metabolic syndrome than those reporting insufficient physical activity; however, the association was not statistically significant after adjustment for age and sex (adjusted OR 0.67, 95% CI 0.35–1.27; *p* = 0.219). The analysis included 242 participants because physical activity could not be classified for 20 participants.

## 4. Discussion

In the present study, we evaluated the classification performance of anthropometric, body composition, and cardiometabolic indices against ALAD-defined metabolic syndrome in Panamanian adults. Composite indices integrating anthropometric and metabolic parameters showed high discriminatory capacity, although their performance varied according to sex. Importantly, several of these indices incorporate variables that overlap with components of the ALAD definition, including waist circumference, triglycerides, and HDL-C. Therefore, the observed AUC values should be interpreted as classification performance relative to the ALAD reference definition rather than as evidence of independent diagnostic ability.

The cardiometabolic index (CMI) demonstrated high discriminatory capacity for identifying ALAD-defined metabolic syndrome, particularly among men, in whom it achieved the highest observed area under the ROC curve (AUC). Furthermore, CMI remained strongly associated with metabolic syndrome after adjustment for age in both the overall population and the sex-stratified analyses. Because CMI incorporates both the waist-to-height ratio (WHtR) and the triglyceride-to-high-density lipoprotein cholesterol ratio (TG/HDL-C), it simultaneously reflects central adiposity and lipid metabolism, two components closely related to metabolic syndrome. This overlap with central adiposity and lipid components of the ALAD definition may partly contribute to its observed classification performance. Our findings are consistent with those reported by Chen et al. [31], who observed a strong association between CMI and metabolic syndrome in a large population-based cohort. Similarly, Wakabayashi et al. [32] reported that CMI values are influenced by sex and age, supporting the need for population-specific evaluation of this index.

The lipid accumulation product (LAP) also demonstrated high discriminatory capacity for identifying ALAD-defined metabolic syndrome. Among women, LAP showed the highest observed area under the ROC curve and remained significantly associated with ALAD-defined metabolic syndrome after adjustment for age. Pairwise comparisons demonstrated that LAP significantly outperformed VAI, TG/HDL-C, and AIP, whereas its classification performance was comparable to that of CMI. LAP combines waist circumference and fasting triglyceride concentration, thereby integrating information on central adiposity and lipid metabolism, two key components of metabolic syndrome. This combined assessment may contribute to its high classification performance. Our findings are consistent with previous studies reporting favorable discriminatory performance of LAP for identifying metabolic syndrome across diverse populations. A recent analysis of the NHANES 2005–2018 cohort likewise identified LAP among the best-performing indices for identifying metabolic syndrome in both men and women [33]. Similarly, Tamini et al. reported that LAP and CMI demonstrated comparable discriminatory performance and outperformed the body adiposity index in adults with obesity [15]. Together, these findings support the robustness and consistency of LAP as a practical marker for identifying metabolic syndrome across different populations.

The triglyceride-to-high-density lipoprotein cholesterol ratio (TG/HDL-C) also demonstrated high discriminatory capacity for identifying ALAD-defined metabolic syndrome and remained significantly associated with the condition after adjustment for age. Because TG/HDL-C reflects the balance between circulating triglycerides and HDL cholesterol, it has been widely recognized as an indicator of atherogenic dyslipidemia and insulin resistance, both of which are closely related to metabolic syndrome. Our findings are consistent with previous studies evaluating TG/HDL-C as a practical marker for identifying metabolic syndrome. Cárdenas-Juárez et al. evaluated TG/HDL-C and other lipid profile ratios in relation to metabolic syndrome diagnosis, providing additional evidence for the potential utility of lipid-derived indices in metabolic syndrome assessment [13]. Likewise, a large population-based study in Korean adults identified TG/HDL-C as a useful marker for identifying metabolic syndrome and established sex-specific cut-off values, supporting its applicability across different populations [34].

The visceral adiposity index (VAI) demonstrated good discriminatory capacity for identifying ALAD-defined metabolic syndrome and remained significantly associated with the condition after adjustment for age in the overall population and in both sex-stratified analyses. Its discriminatory performance nevertheless varied by sex, with a higher AUC in men than in women. Unlike conventional anthropometric measures, VAI combines waist circumference, body mass index, triglycerides, and HDL cholesterol to provide an indirect estimate of visceral adipose tissue dysfunction, thereby integrating both anthropometric and metabolic information. This multidimensional approach may explain its association with several cardiometabolic disorders beyond measures of general obesity. Our findings are consistent with the concept originally proposed by Amato et al., who developed VAI as an indirect indicator of visceral adipose tissue dysfunction and cardiometabolic risk [35]. More recently, Jakubiak et al. summarized the growing evidence supporting the association of VAI with metabolic syndrome, insulin resistance, type 2 diabetes, metabolic dysfunction-associated steatotic liver disease, cardiovascular disease, and chronic kidney disease, while emphasizing the need for further validation studies [9]. The sex-specific differences observed in our study further support the importance of evaluating VAI according to both sex and population characteristics rather than assuming uniform classification performance across different populations.

The waist-to-height ratio (WHtR) also demonstrated high discriminatory capacity for identifying ALAD-defined metabolic syndrome and remained significantly associated with the condition after adjustment for age in both the overall population and the sex-stratified analyses. Because WHtR standardizes waist circumference according to height, it provides a simple measure of central adiposity that is closely related to metabolic syndrome. Our findings are consistent with previous evidence supporting WHtR as one of the most useful anthropometric indices for identifying metabolic syndrome and other cardiometabolic disorders. A systematic review and meta-analysis by Ashwell et al. demonstrated that WHtR consistently outperformed both body mass index and waist circumference for detecting cardiometabolic risk factors across multiple ethnic populations [36]. More recently, Chan et al. reported that WHtR showed better performance than body mass index and waist circumference for screening metabolic syndrome in older adults, while noting that additional studies are needed to determine appropriate WHtR cut-off values in this population [37]. These findings support the potential utility of WHtR as a simple anthropometric measure for metabolic syndrome screening [37].

Body mass index (BMI) also demonstrated good discriminatory capacity for identifying ALAD-defined metabolic syndrome and remained significantly associated with the condition after adjustment for age in both the overall population and the sex-stratified analyses. Despite its widespread use as a measure of general adiposity, BMI does not distinguish between fat mass and lean mass or provide information on fat distribution, which may limit its ability to identify individuals with metabolic abnormalities associated with central adiposity. These limitations may explain why composite indices that incorporate measures of central adiposity and metabolic abnormalities, such as CMI and LAP, showed higher observed discriminatory performance in our study. Nevertheless, BMI remains a practical and widely available screening tool because of its simplicity and routine availability. Recent evidence suggests that BMI should not be used as a standalone measure of obesity but rather in combination with indicators of fat distribution, such as waist circumference or WHtR, to improve the assessment of adiposity and obesity-related health risk, which is consistent with our findings [38].

Pairwise comparisons of ROC curves using the DeLong test provided additional insight into the relative classification performance of the evaluated indices. In men, CMI showed significantly greater classification performance than BMI, TG/HDL-C, and AIP, whereas no statistically significant differences were observed between CMI and the remaining indices. In women, LAP showed significantly greater classification performance than VAI, TG/HDL-C, and AIP, whereas no statistically significant differences were observed between LAP and the remaining indices. Thus, although CMI in men and LAP in women showed the highest observed AUCs, statistical superiority was demonstrated only for the specific pairwise comparisons supported by the DeLong test. Notably, no statistically significant difference was observed between LAP and HOMA-IR, suggesting that simple indices derived from routinely available anthropometric and biochemical measurements may have potential utility for identifying ALAD-defined metabolic syndrome.

The atherogenic index of plasma (AIP) also demonstrated good discriminatory capacity for identifying ALAD-defined metabolic syndrome and remained significantly associated with the condition after adjustment for age. AIP, calculated as the logarithmic transformation of the triglyceride-to-HDL cholesterol ratio, has been proposed as an indirect marker of atherogenic dyslipidemia and cardiovascular risk because it reflects the balance between triglyceride-rich lipoproteins and protective HDL cholesterol. Although AIP performed well in our study, its discriminatory capacity was lower than that of CMI and LAP and did not exceed that of the original TG/HDL-C ratio. These findings suggest that, in our study population, the logarithmic transformation of the TG/HDL-C ratio did not provide additional classification value for identifying ALAD-defined metabolic syndrome. This interpretation is consistent with recent evidence demonstrating that AIP is significantly associated with metabolic syndrome and several cardiometabolic disorders, although its clinical utility varies according to the population and outcome evaluated, supporting its role as a useful, although not consistently superior, cardiometabolic marker [39].

An additional analysis evaluated the association between self-reported physical activity and ALAD-defined metabolic syndrome. Although participants reporting sufficient physical activity had lower estimated odds of metabolic syndrome than those reporting insufficient physical activity, the association was not statistically significant after adjustment for age and sex (adjusted OR 0.67, 95% CI 0.35–1.27; *p* = 0.219). These findings should be interpreted cautiously because physical activity was self-reported and could not be classified for 20 participants. In addition, the cross-sectional design precludes establishing temporal or causal relationships between physical activity and metabolic syndrome.

Because the evaluated cardiometabolic indices are derived from routinely collected anthropometric measurements and standard biochemical tests, they can be easily calculated using information already available during primary care consultations and nutritional assessments. This practical advantage may facilitate the identification of individuals with ALAD-defined MetS without requiring additional laboratory testing or specialized diagnostic procedures.

Beyond facilitating the identification of ALAD-defined MetS, early recognition of individuals with an adverse cardiometabolic profile may also facilitate the implementation of preventive strategies before the development of long-term complications. MetS is a complex multisystem disorder associated with an increased risk of type 2 diabetes, metabolic dysfunction-associated steatotic liver disease (MASLD), chronic kidney disease, and cardiovascular disease through interconnected mechanisms involving insulin resistance, visceral adiposity, chronic low-grade inflammation, endothelial dysfunction, and dyslipidemia [40]. Within the cardiovascular spectrum, TG/HDL-C has also been associated with clinically relevant outcomes; Rosca et al. recently reported an association between TG/HDL-C and ischemic stroke risk in patients with paroxysmal atrial fibrillation [41]. Therefore, simple and accessible cardiometabolic indices may have clinical utility not only for identifying ALAD-defined MetS but also for supporting early cardiometabolic risk assessment in routine clinical practice.

This study has several strengths. To our knowledge, it is the first study to comprehensively evaluate and compare the classification performance of multiple anthropometric, body composition, and cardiometabolic indices for identifying ALAD-defined metabolic syndrome in Panamanian adults without diabetes. Unlike previous studies that evaluated only a limited number of indices, the present study compared anthropometric, body composition, and cardiometabolic indices within the same study population using a standardized analytical framework. The analytical approach included age-adjusted logistic regression models, sex-stratified analyses, receiver operating characteristic curve analyses, optimal cut-off determination, and formal pairwise comparisons using DeLong’s test, providing a comparative assessment of the relative classification performance of the evaluated indices. Furthermore, the use of the ALAD definition enhances the clinical relevance of our findings for Latin American populations, where body composition and cardiometabolic risk profiles may differ from those reported in other regions.

Several limitations should be considered when interpreting these findings. First, the cross-sectional design precludes assessment of temporal relationships or prediction of future cardiometabolic outcomes. Second, because several evaluated indices incorporate anthropometric and biochemical variables that are also components of the ALAD definition of metabolic syndrome, their classification performance may be partly influenced by mathematical overlap with the reference definition. Accordingly, the observed AUCs should not be interpreted as evidence of independent diagnostic ability. Third, the sex-specific cut-off values were derived from the same study population and should therefore be considered exploratory. Although internal validation using 2000 stratified bootstrap resamples was performed to assess their stability and uncertainty, bootstrap validation does not replace external validation in an independent population. The relatively small number of participants with metabolic syndrome in the sex-stratified analyses (27 men and 24 women) also limits the precision and stability of the estimated cut-offs, sensitivity, and specificity, as reflected by the variability of the bootstrap estimates. Although complementary models additionally adjusted for sex and physical activity yielded results consistent with the primary analyses, further multivariable adjustment was limited by the number of metabolic syndrome events, particularly in the complete-case HOMA-IR analysis. Accordingly, these complementary models should be interpreted as exploratory, and residual confounding by other unmeasured or incompletely characterized clinical and lifestyle factors cannot be excluded. In addition, pairwise DeLong comparisons were not adjusted for multiple testing; therefore, the results of these pairwise comparisons should be interpreted as exploratory rather than confirmatory. Overall, composite indices integrating anthropometric and metabolic parameters, particularly CMI and LAP, showed high discriminatory capacity for identifying ALAD-defined metabolic syndrome in Panamanian adults without diabetes. Although conventional anthropometric measures such as WHtR and BMI also showed good classification performance, indices incorporating both central adiposity and lipid metabolism showed the highest observed classification performance.

Because these indices are derived from simple anthropometric measurements and routine biochemical tests, they may represent practical and inexpensive tools for identifying individuals with prevalent ALAD-defined metabolic syndrome, particularly in primary healthcare and resource-limited settings. Future studies should externally validate these findings and the proposed cut-off values in independent Panamanian and Latin American populations and evaluate their ability to predict incident metabolic syndrome and other long-term cardiometabolic outcomes.

## 5. Conclusions

In conclusion, composite indices integrating anthropometric and metabolic parameters, particularly CMI and LAP, showed the highest observed classification performance for identifying ALAD-defined metabolic syndrome in Panamanian adults without diabetes. Although conventional anthropometric measures such as WHtR and BMI also performed well, indices incorporating both central adiposity and lipid metabolism generally showed higher observed discriminatory capacity, a difference that reached statistical significance in some, but not all, pairwise comparisons. Given their simplicity, low cost, and reliance on routinely available anthropometric and biochemical measurements, these indices may represent practical tools for screening ALAD-defined metabolic syndrome in primary healthcare settings. Further validation of these findings and the proposed cut-off values in independent Panamanian and Latin American populations is warranted before widespread clinical adoption.

## Figures and Tables

**Table 1:** ALAD criteria used for metabolic syndrome classification.

Component	Criterion
Central obesity	Waist circumference ≥ 94 cm (men); ≥88 cm (women)
Triglycerides	≥150 mg/dL
HDL-C	<40 mg/dL (men); <50 mg/dL (women)
Blood pressure	≥130 mmHg systolic and/or ≥85 mmHg diastolic or current antihypertensive treatment
Fasting plasma glucose	≥100 mg/dL

Abbreviations: HDL-C, high-density lipoprotein cholesterol [18].

**Table 2:** Anthropometric, body composition, and cardiometabolic indices evaluated in the study.

Index	Abbreviation	Formula	Original Reference
Homeostasis Model Assessment of Insulin Resistance	HOMA-IR	(Fasting insulin (µU/mL) × Fasting plasma glucose (mg/dL))/405	[23]
Triglyceride-to-HDL Cholesterol Ratio	TG/HDL-C	Triglycerides (mg/dL)/HDL-C (mg/dL)	[24]
Atherogenic Index of Plasma	AIP	log10 [Triglycerides (mmol/L)/HDL-C (mmol/L)]	[25]
Waist-to-Height Ratio	WHtR	Waist circumference (cm)/Height (cm)	[26]
Cardiometabolic Index	CMI	WHtR × [Triglycerides (mmol/L)/HDL-C (mmol/L)]	[27]
Lipid Accumulation Product	LAP	Men: (Waist circumference [cm] − 65) × Triglycerides (mmol/L)	[28]
		Women: (Waist circumference [cm] − 58) × Triglycerides (mmol/L)	
Visceral Adiposity Index	VAI	Men: [WC/(39.68 + 1.88 × BMI)] × (TG/1.03) × (1.31/HDL-C)	[29]
		Women: [WC/(36.58 + 1.89 × BMI)] × (TG/0.81) × (1.52/HDL-C)	
Visceral Fat Rating	VFR	Estimated by multi-frequency bioelectrical impedance analysis using the Tanita MC-580 proprietary algorithm	[30]

Abbreviations: HOMA-IR, Homeostasis Model Assessment of Insulin Resistance; TG/HDL-C, triglyceride-to-high-density lipoprotein cholesterol ratio; AIP, atherogenic index of plasma; WHtR, waist-to-height ratio; CMI, cardiometabolic index; LAP, lipid accumulation product; VAI, visceral adiposity index; VFR, visceral fat rating; WC, waist circumference; TG, triglycerides; HDL-C, high-density lipoprotein cholesterol.

**Table 3:** Baseline characteristics of the study population according to sex and metabolic syndrome status.

Variable	Men Without MetS (n = 83)	Men with MetS (n = 27)	*p* Value	Women Without MetS (n = 128)	Women with MetS (n = 24)	*p* Value
Age (years)	36.0 (25.0–44.8)	40.0 (31.0–48.5)	0.109	39.0 (25.0–46.0)	43.0 (34.5–49.0)	0.043
Anthropometric and body composition measures						
Weight (kg)	78.8 (71.1–88.0)	94.3 (87.0–109.4)	<0.001	68.0 (59.5–80.0)	90.0 (73.0–99.6)	<0.001
Height (cm)	173.0 (170.0–176.8)	170.5 (166.8–176.0)	0.216	159.0 (156.0–164.2)	158.9 (155.9–162.0)	0.374
BMI (kg/m^2^)	26.2 (23.6–29.4)	33.3 (29.9–37.5)	<0.001	27.1 (23.6–30.7)	35.4 (30.0–39.3)	<0.001
Waist circumference (cm)	89.5 (83.0–97.0)	103.5 (100.8–120.0)	<0.001	85.0 (75.9–98.0)	102.5 (94.0–110.8)	<0.001
Neck circumference (cm)	39.0 (37.0–41.0)	42.7 (41.0–44.3)	<0.001	33.0 (31.0–35.6)	37.0 (36.0–40.0)	<0.001
WHtR	0.52 (0.48–0.57)	0.63 (0.58–0.69)	<0.001	0.54 (0.47–0.60)	0.66 (0.59–0.70)	<0.001
Visceral Fat Rating (VFR)	9 (6–11)	14 (12–18)	<0.001	6 (4–8)	11 (8–12)	<0.001
Blood pressure (mmHg)						
Systolic blood pressure	124.0 (118.0–130.5)	131.0 (127.0–137.0)	<0.001	115.2 (108.0–122.0)	130.5 (124.8–142.5)	<0.001
Diastolic blood pressure	79.0 (72.8–81.8)	85.0 (78.0–89.0)	0.006	73.0 (69.0–79.6)	84.0 (80.8–90.0)	<0.001
Biochemical measurements						
Fasting glucose (mg/dL)	89.0 (85.0–94.0)	98.0 (91.0–102.5)	<0.001	88.0 (83.0–92.0)	91.0 (84.8–101.0)	0.058
HbA1c (%)	5.5 (5.3–5.8)	5.6 (5.4–6.0)	0.095	5.5 (5.3–5.7)	5.6 (5.4–5.9)	0.075
Total cholesterol (mg/dL)	180.0 (160.5–220.0)	192.0 (164.5–223.0)	0.479	199.5 (165.0–224.2)	203.5 (177.8–231.5)	0.539
LDL-C (mg/dL)	114.8 (92.5–145.8)	123.4 (90.8–142.8)	0.803	118.2 (87.8–147.6)	136.8 (101.0–156.6)	0.158
HDL-C (mg/dL)	48.0 (41.0–59.0)	37.0 (32.0–40.5)	<0.001	56.5 (46.8–67.2)	43.0 (36.0–47.0)	<0.001
Triglycerides (mg/dL)	83.0 (54.5–127.5)	171.0 (120.0–237.5)	<0.001	86.5 (56.8–128.0)	138.5 (89.5–185.2)	<0.001
Fasting insulin (µU/mL) †	12.1 (8.2–16.4)	18.4 (15.4–23.9)	<0.001	12.4 (8.7–17.7)	24.5 (20.7–29.3)	<0.001
Cardiometabolic indices						
HOMA-IR †	2.71 (1.84–3.82)	4.08 (3.77–4.93)	0.002	2.64 (1.96–3.78)	5.38 (4.79–6.95)	<0.001
TG/HDL-C	1.82 (1.09–2.56)	4.27 (3.07–7.15)	<0.001	1.54 (0.97–2.62)	3.19 (2.20–4.60)	<0.001
AIP	−0.100 (−0.323–0.048)	0.271 (0.128–0.493)	<0.001	−0.173 (−0.375–0.058)	0.143 (−0.018–0.302)	<0.001
LAP	27.1 (11.2–41.4)	76.0 (55.9–137.7)	<0.001	25.0 (13.3–47.9)	73.8 (52.5–88.5)	<0.001
CMI	0.90 (0.49–1.37)	2.50 (1.95–4.46)	<0.001	0.75 (0.48–1.55)	2.19 (1.42–2.91)	<0.001
VAI	0.96 (0.58–1.40)	2.65 (1.85–4.20)	<0.001	1.17 (0.77–2.08)	2.86 (1.80–3.56)	<0.001

Continuous variables are presented as median (interquartile range). Comparisons between participants with and without metabolic syndrome were performed separately for each sex using the Mann–Whitney U test. MetS, metabolic syndrome; BMI, body mass index; WHtR, waist-to-height ratio; VFR, visceral fat rating; SBP, systolic blood pressure; DBP, diastolic blood pressure; HbA1c, glycated hemoglobin; LDL-C, low-density lipoprotein cholesterol; HDL-C, high-density lipoprotein cholesterol; HOMA-IR, Homeostasis Model Assessment of Insulin Resistance; TG/HDL-C, triglyceride-to-high-density lipoprotein cholesterol ratio; AIP, atherogenic index of plasma; LAP, lipid accumulation product; CMI, cardiometabolic index; VAI, visceral adiposity index. † Fasting insulin and HOMA-IR were available for fewer participants. Sample size varied slightly according to the availability of data for each variable.

**Table 4:** Classification performance of anthropometric, body composition, and cardiometabolic indices for identifying ALAD-defined metabolic syndrome.

Index	n	AUC (95% CI)	Optimal Cut-Off	Sensitivity (%)	Specificity (%)	PPV (%)	NPV (%)
Men							
CMI	105	0.933 (0.887–0.979)	1.52	92.6	83.3	65.8	97
LAP	105	0.922 (0.868–0.975)	44.44	92.6	82.1	64.1	97
TG/HDL-C	110	0.887 (0.821–0.953)	2.61	88.9	77.1	55.8	95.5
VAI	105	0.908 (0.847–0.968)	1.76	85.2	84.6	65.7	94.3
AIP	110	0.887 (0.821–0.953)	0.057	88.9	77.1	55.8	95.5
WHtR	105	0.880 (0.817–0.943)	0.56	92.6	71.8	53.2	96.6
BMI	105	0.807 (0.706–0.909)	29.6	77.8	76.9	53.8	90.9
VFR	107	0.841 (0.767–0.915)	11	92.6	66.2	48.1	96.4
HOMA-IR	55	0.788 (0.669–0.906)	3.13	100	57.1	41.9	100
Women							
CMI	150	0.837 (0.752–0.923)	1.04	91.7	66.7	34.4	97.7
LAP	151	0.854 (0.778–0.931)	33.59	95.8	63	32.9	98.8
TG/HDL-C	152	0.804 (0.714–0.895)	1.85	87.5	64.8	31.8	96.5
VAI	149	0.809 (0.721–0.898)	1.27	95.8	58.4	30.7	98.6
AIP	152	0.804 (0.714–0.895)	−0.093	87.5	64.8	31.8	96.5
WHtR	150	0.848 (0.781–0.915)	0.56	100	65.1	35.3	100
BMI	149	0.812 (0.722–0.901)	29.62	83.3	71.2	35.7	95.7
VFR	143	0.847 (0.783–0.911)	7	100	63	35.3	100
HOMA-IR	83	0.847 (0.727–0.968)	4.27	83.3	80.3	41.7	96.6

Classification performance was evaluated using ALAD-defined metabolic syndrome as the reference standard. AUC, area under the receiver operating characteristic curve; CI, confidence interval; PPV, positive predictive value; NPV, negative predictive value; CMI, cardiometabolic index; LAP, lipid accumulation product; TG/HDL-C, triglyceride-to-high-density lipoprotein cholesterol ratio; VAI, visceral adiposity index; AIP, atherogenic index of plasma; WHtR, waist-to-height ratio; BMI, body mass index; VFR, visceral fat rating; HOMA-IR, homeostasis model assessment of insulin resistance. HOMA-IR analyses were restricted to participants with available fasting insulin measurements. Sample size varied according to the availability of the variables required to calculate each index.

**Table 5:** Pairwise comparisons of areas under correlated ROC curves using the DeLong test.

Comparison	Difference in AUC	*p* Value
Men		
CMI vs. LAP	0.011	0.473
CMI vs. VAI	0.025	0.105
CMI vs. TG/HDL-C	0.033	0.032
CMI vs. AIP	0.033	0.032
CMI vs. WHtR	0.053	0.141
CMI vs. VFR	0.078	0.056
CMI vs. BMI	0.126	0.023
CMI vs. HOMA-IR	0.087	0.207
Women		
LAP vs. WHtR	0.011	0.762
LAP vs. VFR	0.023	0.531
LAP vs. HOMA-IR	0.03	0.665
LAP vs. CMI	0.022	0.193
LAP vs. BMI	0.049	0.366
LAP vs. VAI	0.052	0.01
LAP vs. TG/HDL-C	0.051	0.027
LAP vs. AIP	0.051	0.027

Differences in AUC were calculated as the AUC of the reference index minus that of the comparator. Positive values indicate a higher observed AUC for the reference index relative to the comparator; statistical significance was determined using the DeLong test. Comparisons involving HOMA-IR were restricted to participants with available fasting insulin measurements. AUC, area under the ROC curve; CMI, cardiometabolic index; LAP, lipid accumulation product; VAI, visceral adiposity index; TG/HDL-C, triglyceride-to-high-density lipoprotein cholesterol ratio; AIP, atherogenic index of plasma; WHtR, waist-to-height ratio; VFR, visceral fat rating; BMI, body mass index; HOMA-IR, homeostasis model assessment of insulin resistance.

**Table 6:** Association between anthropometric, body composition, and cardiometabolic indices and ALAD-defined metabolic syndrome in age-adjusted logistic regression models.

Index	Overall OR (95% CI)	*p* Value	Men OR	*p* Value	Women OR (95% CI)	*p* Value
			(95% CI)			
CMI	4.84 (3.03–7.76)	<0.001	8.39 (3.22–21.88)	<0.001	3.67 (2.09–6.47)	<0.001
LAP †	1.61 (1.40–1.86)	<0.001	1.98 (1.47–2.68)	<0.001	1.47 (1.24–1.74)	<0.001
TG/HDL-C	2.14 (1.68–2.72)	<0.001	2.34 (1.61–3.42)	<0.001	1.95 (1.40–2.71)	<0.001
AIP ‡	1.72 (1.45–2.03)	<0.001	1.99 (1.49–2.66)	<0.001	1.53 (1.24–1.89)	<0.001
WHtR ‡	4.88 (3.01–7.92)	<0.001	5.97 (2.73–13.04)	<0.001	4.94 (2.47–9.90)	<0.001
BMI	1.18 (1.11–1.25)	<0.001	1.17 (1.08–1.28)	<0.001	1.20 (1.10–1.30)	<0.001
VFR	1.29 (1.18–1.40)	<0.001	1.34 (1.17–1.53)	<0.001	1.31 (1.14–1.50)	<0.001
VAI	2.94 (2.11–4.08)	<0.001	7.19 (2.99–17.27)	<0.001	2.37 (1.57–3.58)	<0.001
HOMA-IR §	1.60 (1.24–2.05)	<0.001	1.64 (1.09–2.47)	0.017	1.60 (1.16–2.22)	0.004

Odds ratios (ORs) and 95% confidence intervals (CIs) were obtained from age-adjusted binary logistic regression models. Each index was evaluated in a separate model. † LAP expressed per 10-unit increment. ‡ AIP and WHtR expressed per 0.1-unit increment. All remaining indices are expressed per 1-unit increment (BMI per 1 kg/m^2^; VFR per 1 rating unit). § HOMA-IR analyses were restricted to participants with available fasting insulin measurements. Because the indices were modeled using different unit scales, the magnitudes of the ORs should not be directly compared across indices.

**Table 7:** Association between physical activity and ALAD-defined metabolic syndrome.

Physical Activity	No MetS n (%)	MetS n (%)	Fisher’s Exact	Adjusted OR (95% CI)	Adjusted
			*p*-Value		*p*-Value
Insufficient	96 (49.7)	29 (59.2)	—	Reference	—
Sufficient	97 (50.3)	20 (40.8)	0.265	0.67 (0.35–1.27)	0.219

Values are column percentages. Fisher’s exact test was used to compare physical activity categories between participants with and without ALAD-defined metabolic syndrome. Adjusted odds ratios (ORs) were estimated using binary logistic regression controlling for age and sex. Physical activity was classified as insufficient (category 1) or sufficient (categories 2–3). Twenty participants were excluded because physical activity could not be classified.

## Data Availability

The data presented in this study are available on request from the corresponding author. The data are not publicly available due to privacy and ethical restrictions related to the protection of participants’ personal and clinical information.

## Supplementary Materials

The following supporting information can be downloaded at: https://www.mdpi.com/article/10.3390/medsci14040500/s1, Table S1: Internal bootstrap validation of sex-specific ROC-derived cut-off values for ALAD-defined metabolic syndrome; Table S2: Complementary multivariable logistic regression analyses for ALAD-defined metabolic syndrome.

## Abbreviations

The following abbreviations are used in this manuscript:

MetS	Metabolic syndrome
ALAD	Latin American Diabetes Association
CMI	Cardiometabolic index
LAP	Lipid accumulation product
VAI	Visceral adiposity index
AIP	Atherogenic index of plasma
TG/HDL-C	Triglyceride-to-high-density lipoprotein cholesterol ratio
WHtR	Waist-to-height ratio
BMI	Body mass index
VFR	Visceral fat rating
HOMA-IR	Homeostasis Model Assessment of Insulin Resistance
ROC	Receiver operating characteristic
AUC	Area under the receiver operating characteristic curve
OR	Odds ratio
CI	Confidence interval
PPV	Positive predictive value
NPV	Negative predictive value
