# Supplementary material for: Comparative Classification Performance of Anthropometric, Body Composition, and Cardiometabolic Indices for Identifying ALAD-Defined Metabolic Syndrome in Panamanian Adults
Source: Med Sci (Basel). 2026 Aug 20;14(4):500. doi: 10.3390/medsci14040500 (PMC13515952; doi:10.3390/medsci14040500)
Supplement: Supplementary file 1 [file medsci-14-00500-s001.zip › medsci-4398808-supplementary.pdf]

Supplementary Table S1. Internal bootstrap validation of sex-specific ROC-derived cut-off values for ALAD-defined metabolic syndrome							
**Sex**	**Index**	**N**	**MetS n**	**Original cut-off**	**Bootstrap cut-off, median (95% CI)**	**Out-of-bag sensitivity, median (95% CI)**	**Out-of-bag specificity, median (95% CI)**
**Men**	CMI	105	27	1.52	1.52 (1.46–1.97)	0.89 (0.54–1.00)	0.83 (0.69–0.96)
	LAP	105	27	44.44	44.44 (44.44–69.11)	0.88 (0.45–1.00)	0.83 (0.70–0.96)
	TG/HDL-C	110	27	2.61	2.77 (2.61–3.88)	0.80 (0.40–1.00)	0.80 (0.62–0.94)
	AIP	110	27	0.06	0.08 (0.06–0.23)	0.80 (0.42–1.00)	0.79 (0.61–0.94)
	WHtR	105	27	0.56	0.56 (0.54–0.60)	0.88 (0.44–1.00)	0.72 (0.52–0.90)
	VAI	105	27	1.76	1.76 (1.09–2.35)	0.78 (0.40–1.00)	0.86 (0.52–1.00)
	BMI	105	27	29.6	29.60 (27.78–31.72)	0.73 (0.36–1.00)	0.75 (0.53–0.91)
	VFR	107	27	11	11.00 (10.00–12.00)	0.90 (0.50–1.00)	0.67 (0.46–0.84)
	HOMA-IR	55	13	3.13	3.34 (3.13–4.07)	0.80 (0.25–1.00)	0.62 (0.39–0.88)
**Women**	CMI	150	24	1.04	1.04 (0.97–2.36)	0.86 (0.29–1.00)	0.67 (0.53–0.94)
	LAP	151	24	33.59	50.63 (33.59–73.58)	0.78 (0.30–1.00)	0.73 (0.53–0.94)
	TG/HDL-C	152	24	1.85	1.85 (1.49–3.27)	0.80 (0.25–1.00)	0.65 (0.45–0.89)
	AIP	152	24	-0.09	-0.09 (-0.19–0.22)	0.80 (0.22–1.00)	0.65 (0.45–0.91)
	WHtR	150	24	0.56	0.56 (0.56–0.63)	1.00 (0.38–1.00)	0.66 (0.55–0.82)
	VAI	149	24	1.27	1.38 (1.27–3.20)	0.88 (0.22–1.00)	0.62 (0.48–0.94)
	BMI	149	24	29.62	29.62 (27.35–33.96)	0.75 (0.33–1.00)	0.71 (0.48–0.89)
	VFR	143	24	7	7.00 (7.00–9.00)	1.00 (0.43–1.00)	0.65 (0.51–0.81)
	HOMA-IR	83	12	4.27	4.96 (2.81–5.47)	0.75 (0.20–1.00)	0.85 (0.59–0.96)

Internal validation was performed using 2,000 bootstrap resamples separately within each sex and stratified by ALAD-defined metabolic syndrome (MetS) status, preserving the number of MetS and non-MetS participants in each resample. For each index, resampling was restricted to participants with available data for that index. The optimal cut-off was re-estimated in each bootstrap sample using the Youden index. Percentile-based 95% confidence intervals describe the distribution of bootstrap cut-off estimates. Sensitivity and specificity were evaluated in out-of-bag observations not selected in each bootstrap resample. All cut-off values should be considered exploratory.

Supplementary Table S2. Complementary multivariable logistic regression analyses for ALAD-defined metabolic syndrome							
**Index**	**n**	**MetS cases**	**Adjusted OR**	**95% CI lower**	**95% CI upper**	**p-value**	**Increment / interpretation**
CMI	236	49	4.93	3.00	8.10	<0.001	per 1-unit increment
LAP	236	49	1.64	1.41	1.91	<0.001	per 10-unit increment
TG/HDL-C	242	49	2.11	1.64	2.72	<0.001	per 1-unit increment
AIP	242	49	1.69	1.42	2.02	<0.001	per 0.1-unit increment
WHtR	236	49	5.45	3.18	9.32	<0.001	per 0.1-unit increment
BMI	235	49	1.19	1.12	1.26	<0.001	per 1 kg/m² increment
VFR	238	49	1.32	1.19	1.45	<0.001	per 1 rating-unit increment
VAI	235	49	3.31	2.25	4.87	<0.001	per 1-unit increment
HOMA-IR	129	23	1.53	1.19	1.98	0.001	per 1-unit increment
**Model specification**							
Outcome	ALAD-defined metabolic syndrome (binary)						
Adjustment	Each index was evaluated in a separate model adjusted for age, sex, and physical activity.						
**Notes**							
1	Complete-case analysis was used for variables required by each model.						
2	Physical activity: insufficient = reference; sufficient = categories 2–3. Sex was included as a covariate in the overall-sample models.						
3	HOMA-IR analysis was restricted to participants with available fasting insulin measurements.						
